# Linear and nonlinear optical properties of hybrid metallic–dielectric plasmonic nanoantennas

**DOI:** 10.3762/bjnano.7.13

**Published:** 2016-01-26

**Authors:** Mario Hentschel, Bernd Metzger, Bastian Knabe, Karsten Buse, Harald Giessen

**Affiliations:** 1 4th Physics Institute and Research Center SCoPE, University of Stuttgart, Pfaffenwaldring 57, 70569 Stuttgart, Germany; 2 Department of Microsystems Engineering, University of Freiburg, Georges-Köhler-Allee 102, 79110 Freiburg, Germany; 3 Fraunhofer Institute for Physical Measurement Techniques IPM, Heidenhofstr. 8, 79110 Freiburg, Germany

**Keywords:** nano-optics, nonlinear optics, plasmonics, second harmonic generation, spectroscopy

## Abstract

We study the linear and nonlinear optical properties of hybrid metallic–dielectric plasmonic gap nanoantennas. Using a two-step-aligned electron beam lithography process, we demonstrate the ability to selectively and reproducibly fill the gap region of nanoantennas with dielectric nanoparticles made of lithium niobate (LiNbO_3_) with high efficiency. The linear optical properties of the antennas are modified due to the large refractive index of the material. This leads to a change in the coupling strength as well as an increase of the effective refractive index of the surrounding. The combination of these two effects causes a red- or blue-shift of the plasmonic modes, respectively. We find that the nonlinear optical properties of the combined system are only modified in the range of one order of magnitude. The observed changes in our experiments in the nonlinear emission can be traced to the changed dielectric environment and thus the modified linear optical properties. The intrinsic nonlinearity of the dielectric used is in fact small when compared to the nonlinearity of the metallic part of the hybrid antennas. Thus, the nonlinear signals generated by the antenna itself are dominant in our experiments. We demonstrate that the well-known nonlinear response of bulk dielectric materials cannot always straightforwardly be used to boost the nonlinear response of nanoscale antenna systems. Our results significantly deepen the understanding of these interesting hybrid systems and offer important guidelines for the design of nanoscale, nonlinear light sources.

## Introduction

The field of plasmonics entails the study of the optical properties of metallic nanoparticles. Collective oscillations of the quasi-free conduction electrons with respect to the fixed ionic background can be excited by an external light field. This displacement of charges leads to strong local electric fields in nanoscale volumes around the nanoparticles. Due to the large resonant dipole moment, which is fundamentally connected to the large number of free conduction electrons, energy can be efficiently channeled from the far-field into the so-called near-field.

As previously discussed [1–2], the strong local fields enable a number of applications and phenomena: The plasmonic resonances in arrangements of multiple nanoparticles can couple together giving rise to collective modes similar to molecular physics [3], which led to the development of the so-called plasmon hybridization model [4]. One can also transport energy on deep subwavelength length scales [5], create the plasmonic analogue of electromagnetically induced transparency (EIT) [6–9], and construct systems with tailorable near-field enhancement and confinement [10–13]. What is more, the resonant behavior of plasmonic particles is partially determined by the refractive index of the surrounding environment [14–16], enabling plasmonic refractive index sensing utilizing ensembles of nanoparticles [17] as well as individual nanostructures [18–20].

It was also realized early on that the enhanced local electric field strength could lead to efficient nonlinear optics in these systems [21] as the radiated intensities scale nonlinearly with the fundamental driving light field.

The first nonlinear optical phenomena in these systems were studied in the beginning of the 1980s. Strong second harmonic generation from metal-island films and microstructured silver films were shown to be related to the enhanced local near-field [22]. Gold and silver nanoparticles in water were shown to enable optical phase conjugation [23–24] with an order of magnitude enhanced optical Kerr coefficient when exciting the particles at their respective plasmon resonance. It was also shown that rough metallic films led to enhanced second-harmonic generation [25–26] as well as to enhanced Raman scattering [27–30] and that both phenomena are related to local field hot spots in the metallic films. The nonlinear optical properties of such composite materials can be modelled by nonlinear extension of the Maxwell–Garnett [31] and effective-medium theories [32–33]. Additionally, difference frequency mixing [34], four wave mixing, second harmonic generation, and other nonlinear optical processes were reported.

In recent years a number of papers and experimental studies have shown the predominant role of the linear optical properties on the nonlinear optical ones. It could be shown that even in systems commonly expected to be governed by so-called “hot spot nonlinearities”, such as gap antennas, the linear response still largely determines the nonlinear light generation [35–39]. This is actually not surprising, as Miller’s rule predicts that the nonlinear conversion is maximum when the linear optical properties exhibit resonances either at the fundamental or the harmonic frequencies [40–43]. To be more specific, the nonlinear conversion takes place largely in the plasmonic material itself, i.e., it is generated by the enhanced fields inside the metallic nanoparticles. From this behavior it can be deduced that the strongly enhanced near-field, within for example nanoscale gaps, play a minor role in the nonlinear conversion process. However, simulations and experiments show a significant enhancement of the field strength in the gap. The reason for this apparent contradiction is in fact obvious: Gold is known to have a large nonlinear susceptibility, much larger than the nonlinear susceptibility of glass, which is usually used as the substrate and therefore the most common material inside or under the nanoscale gap [40,44]. Consequently, no enhanced nonlinear signal is expected unless the increasing field strength within the gap causes increased field strength within the adjacent gold as well. Published data shows that this process only starts to play a significant role for gap sizes on the order of 20 nm or less [37]. However, a number of recent publications show a strong influence of the gap size of nanoantennas or rough surfaces on nonlinear processes. In these cases, the antenna itself is not the source of the signal, but rather an optically active species is responsible and the antenna is “dark”. This observation is in particular true for surface-enhanced Raman scattering (SERS) [45–47] and for experiments on surface-enhanced infrared absorption spectroscopy (SEIRA) [48–49]. If one indeed aims at mapping the near-field, one can make use of two-photon photoluminescence [50–51], multiphoton absorption [52], selectively placed molecules for SEIRA [53], or by directly measuring them by scattering and scanning type near-field optical microscopy [54–56]. In these experiments strongly enhanced fields within the gap have been found as well.

In order to benefit from these strongly enhanced fields, a nonlinear optical medium should be placed into the nanoscale gap, or at least within the enhanced near-field in the vicinity of the nanostructure. Even though this combination of field enhancement and nonlinear optics has already been proposed in the first publications on metamaterials and plasmonics [57], only a very limited number of papers report conclusive experiments that are well supported by data [58–68]. In most experiments the role of the plasmonic structures is twofold in that it concentrates the incoming radiation and it is the source of the harmonic radiation. In 2007 Chen and co-workers demonstrated plasmon-enhanced second harmonic generation from ionic self-assembled multilayer films [58]. The authors utilized silver triangles fabricated by colloidal lithography to enhance the second harmonic emission from 3 bilayers of the ionic self-assembled film by about a factor of 1600. In 2008 Kim et al. [59] reported high-harmonic generation when argon gas was blown on bowtie antenna arrays. However, recent results reported by Sivis et al. [69] proved that the observed phenomenon is in fact connected to enhanced atomic line emission rather than higher harmonic generation. Another very convincing experiment was performed by Niesler and co-workers in 2009 [60]. The authors fabricated split-ring resonators on top of a crystalline gallium arsenide substrate and have demonstrated enhanced second harmonic emission caused by the interplay of the local near-field of the split-ring resonator and the substrate. Recently, Alu and Belkin have reported similar results in the mid-infrared spectral region [70]. As a last example, Utikal et al. [61] buried gold gratings within dielectric waveguides consisting of alumina, indium tin oxide, or tungsten trioxide, respectively, and studied the third harmonic spectra. They found that the overall signal is generated not only by the gold itself, but by the dielectric waveguide as well, depending on its microscopic nonlinearity.

## Results and Discussion

**Figure 1:**
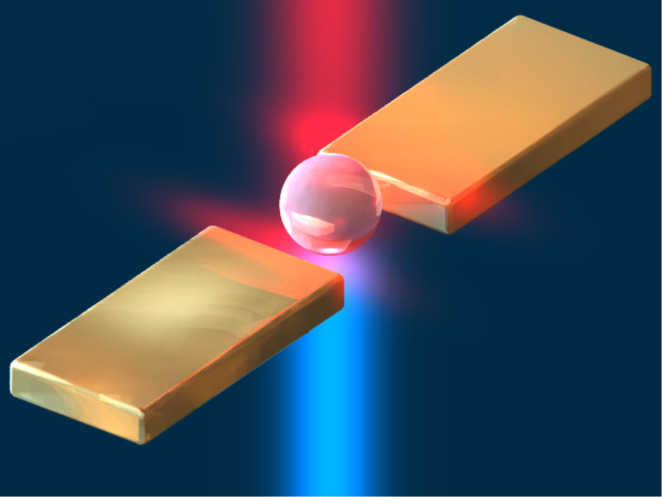
Artists impression of a gold nanoantenna loaded with a nonlinear optical active material. The nanoantenna confines the incoming radiation, enhancing its field strength significantly in the process. The local fields then drive the conversion process in the nonlinear material. If the material intrinsically breaks inversion symmetry, it can produce even numbered harmonics. These even orders cannot be generated by the antenna itself, due to its centrosymmetry, at least as long as one only considers the electric dipole approximation. Reproduced with permission from [65], Copyright (2014) American Chemical Society.

One obvious idea drawn from these earlier experiments is thus to use standard nonlinear materials such as lithium niobate crystals (LiNbO_3_) or indium tin oxide (ITO) and selectively position them inside the gaps of nanoantennas, as shown in an artist’s impression in Figure 1. These materials can be fabricated as nanoparticles, either directly from a wet chemical process or by mechanical milling [71–72].

Another benefit afforded by the nanocrystal/nanoantenna array approach is as follows: As the nanoantenna array is inversion symmetric, in first-order approximation it will not exhibit a second harmonic response for a normal incident electric field. The LiNbO_3_ nanocrystals, however, intrinsically break the inversion symmetry due to their crystal structure. The key idea in combining these two systems is thus to boost only the second harmonic response from the nanocrystals while the antenna array itself remains “dark” (meaning it does not cause any second harmonic light).

**Figure 2:**
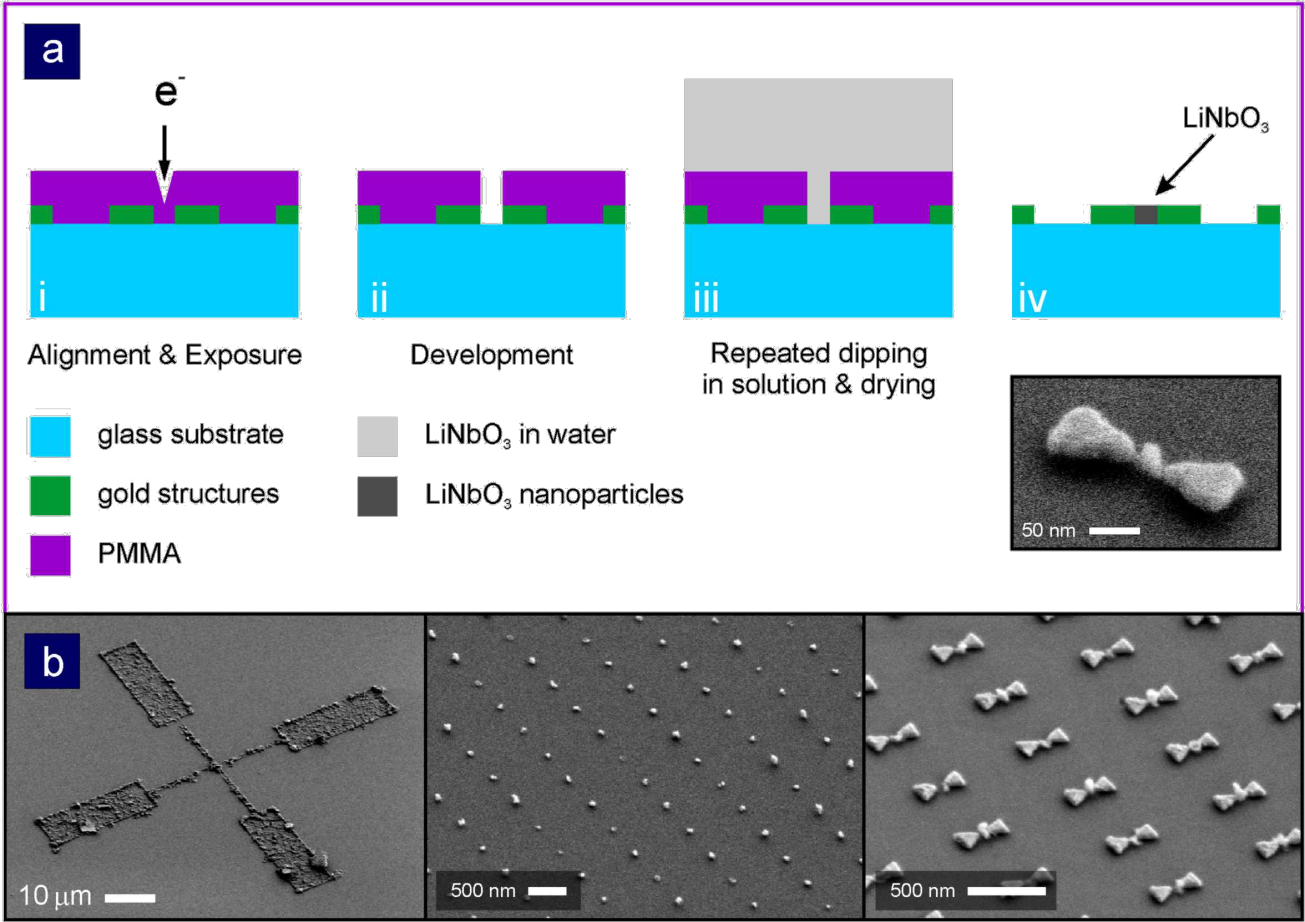
(a) Production steps for the selective filling of gap nanoantennas with LiNbO_3_ nanocrystals. (i) In the first step a substrate with gold nanoantennas and alignment marks is covered with PMMA. After careful alignment, openings are defined within the resist layer directly above the gap regions of the antennas. (ii) The sample is developed and oxygen-plasma cleaned. (iii) The sample is dipped into a solution of LiNbO_3_ nanocrystals and water and afterwards blown dry using nitrogen. This routine is repeated until additionally defined large openings appear to be filled (optical microscope inspection), see first image in (b). (iv) After lift-off, only the LiNbO_3_ nanocrystals deposited inside the antenna gaps remain. Right bottom picture in (a) is a close-up SEM micrograph of a single structure. (b) Overview SEM micrographs of fabricated structures (after lift-off). Left: Cross-mark consisting of nanocrystals. Middle: Reference array of LiNbO_3_ nanocrystals. Right: Selectively filled, LiNbO_3_ nanocrystal, bowtie nanoantenna array. Figure adapted from [2].

Figure 2a illustrates the basic steps in producing these samples. Gold nanoantennas as well as gold alignment marks are fabricated via standard electron beam lithography in poly(methyl methacrylate) (PMMA) resist on a fused silica substrate (suprasil, Heraeus), followed by evaporation of a chromium adhesion and a gold layer, and a subsequent lift-off procedure. The sample is again coated afterward with PMMA. Using the alignment marks, openings are created in the resist that expose the gap regions of the antennas. After development and oxygen plasma cleaning, the samples are immersed in the LiNbO_3_ solution, which consists of the nanocrystals diluted in water. The sample is repeatedly dipped into the solution and afterwards blown dry using nitrogen. The crystals are highly hydrophobic, as is the PMMA layer. However, the particles seem to be strongly attracted to the bare glass surface. Therefore, they agglomerate on the exposed glass surface. Additionally, there is a strong attraction between the LiNbO_3_ nanocrystals such that clusters of particles form and grow with every additional dipping step. As a final step the PMMA layer is removed in acetone. During this step the sample is rested upside down on additional pieces of glass in order to prevent the nanocrystals on top of the PMMA layer from migrating onto the substrate. The small inset in Figure 2a depicts a tilted-view SEM micrograph of a single gold bowtie nanoantenna. One can clearly see the nanocrystals that have agglomerated in the gap region.

In Figure 2b, additional SEM micrographs of the fabricated structures are shown. In order to track the agglomeration using an optical microscope, large crosses are defined in the resist layer. Within these openings the particles will accumulate as well. In contrast to the small openings, the intentional filling of these large crosses can be easily monitored. The SEM micrograph depicts such a cross after lift-off. The residual structures thus consist solely of LiNbO_3_ nanocrystals. The next image shows a reference structure. No antenna structures have been defined, yet with the same periodicity with the same sized openings have been defined. After depositing the particles, one observes a perfect square lattice of LiNbO_3_ nanocrystals. In particular, no defects or empty lattice sides can be found, indicating the extremely high efficiency of this easy and straightforward process. The last image depicts an array of selectively filled bowtie antennas. Again, every antenna is filled with LiNbO_3_ nanocrystals. Most importantly, one can see that no particles are deposited in between the antennas.

**Figure 3:**
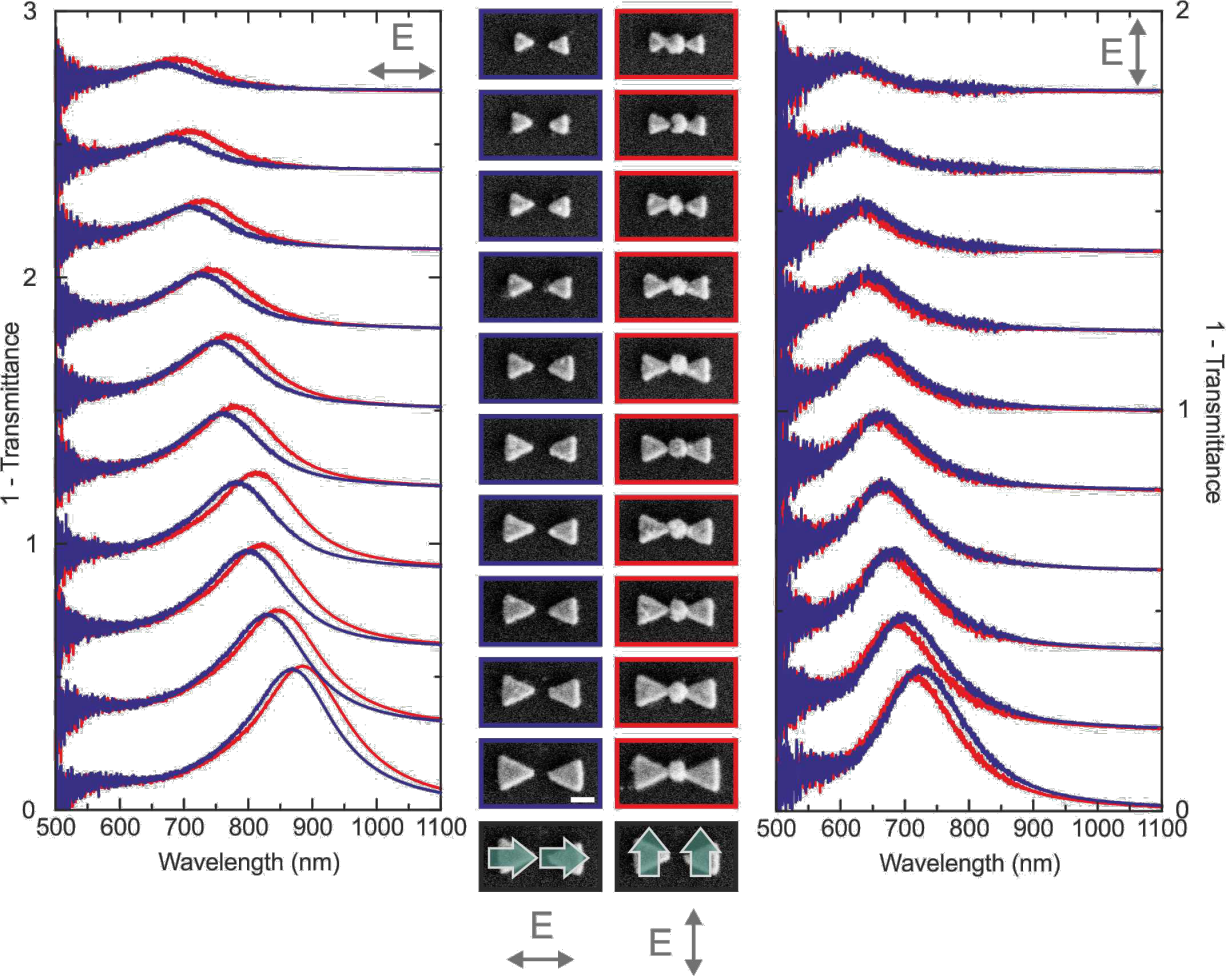
Linear extinction (1 − *T*) spectra for bowtie antenna arrays (blue) and bowtie antennas selectively filled with lithium niobate (red) for excitation polarized along the antenna axis (left column) and perpendicular to the antenna axis (right column). The antenna gap is fixed at ≈50 nm, the basis size of the antennas is increased from top to bottom. The linear spectra for both polarization directions exhibit a red-shift of the plasmonic modes with increasing size, as expected. Inserting the lithium niobate nanoparticles into the gap increases the coupling strength between the two antenna arms. Due to the different mode character (as sketched in the bottom of the figure where green arrows indicate the plasmonic dipoles), the modes exhibit a different spectral shift. For excitation along the antenna axis, the attractive interaction between the particle plasmon is increased, leading to a red-shift. For excitation perpendicular to the antenna axis the repulsive interaction between the dipoles causes a blue-shift. Overall, the spectra demonstrate the excellent filling rate of the antenna gaps, leading to a consistent behavior between the different arrays. Figure adapted from [2].

In Figure 3 we display the linear optical properties of bowtie nanoantennas that have been selectively filled with LiNbO_3_ nanocrystals. The basic size of the triangles has been varied in order to shift the position of the linear resonance over the entire accessible wavelength range of the laser sources, while the gap size has been kept constant. The blue spectra depict the response of the empty bowtie antennas. For excitation along the antenna axis (left column), the strong dipolar resonance undergoes a significant red-shift with increasing size in addition to an increase in the scattering amplitude. This is expected due to the increasing size and volume of the nanostructure. For excitation perpendicular to the antenna axis (right column), one observes a resonance as well, yet due to the smaller dimensions of the antennas in this direction, the resonance is significantly blue-shifted.

The red spectra in Figure 3 show the optical response of the LiNbO_3_-filled antennas. For all geometries, one observes spectral shifts in the position of the resonances, indicating that the antenna gaps have been filled with high efficiency. On closer inspection, one observes a red-shift for excitation along the antenna axis and a blue-shift for excitation perpendicular to the antenna axis. The reason for this behavior is the different character of the plasmonic modes. In both cases, they are hybridized modes between the two dipolar modes of the individual triangles. Yet, for excitation along the antenna axis, it is a head-to-tail configuration, whereas for excitation perpendicular to the axis, one observes a head-to-head configuration (see sketches with green arrows in Figure 3). Filling the antenna gap with a high-refractive-index material leads to an increased coupling strength between the two triangles. For the head-to-tail configuration this results in a lowering of the resonance energy due to increased attractive interaction. For the head-to-head configuration, in contrast, the repulsive interaction will increase and therefore cause a blue shift of the mode. Overall, the spectra demonstrate the excellent filling rate, manifesting itself in pronounced and reproducible spectral shifts in the linear response.

**Figure 4:**
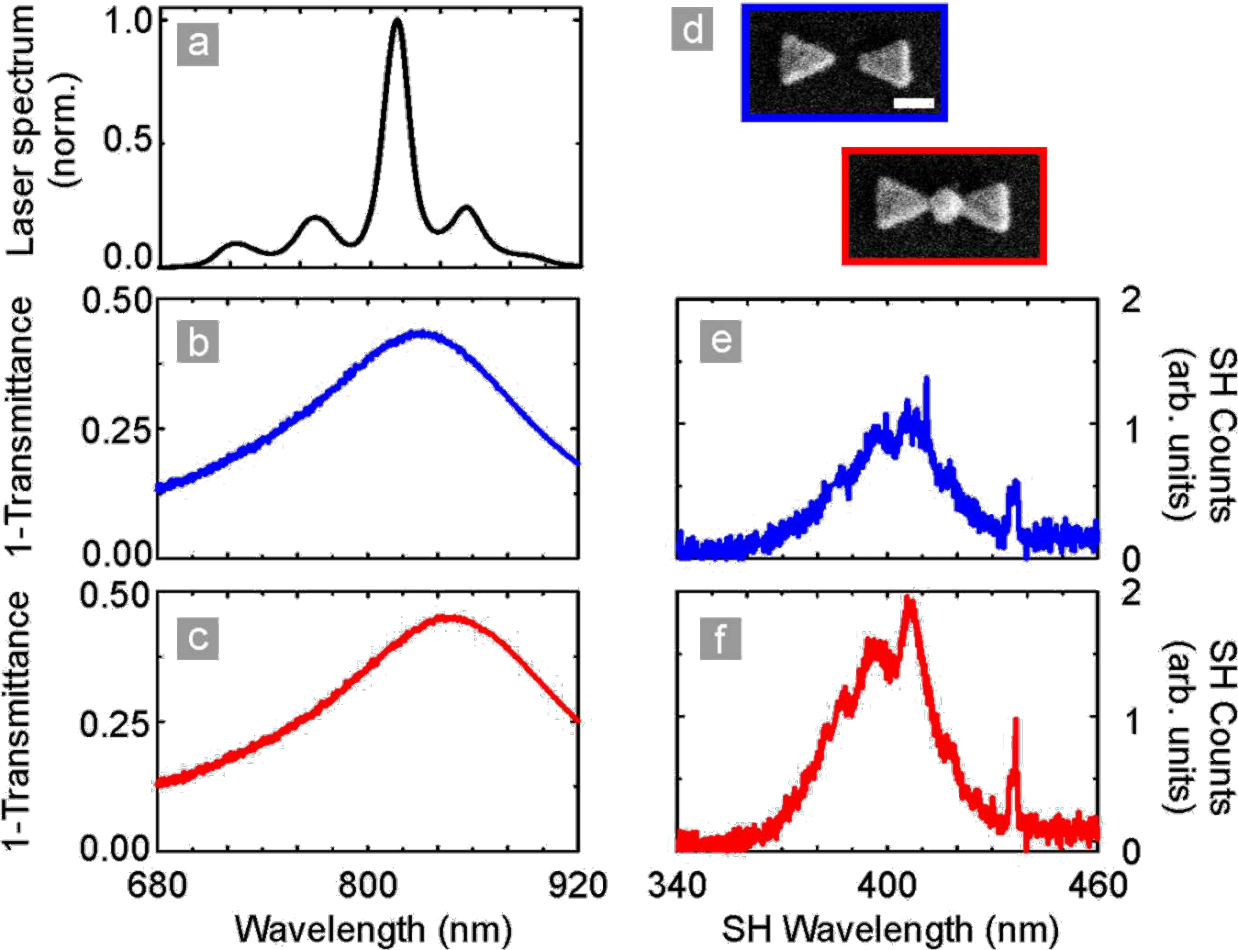
Linear and nonlinear properties of a bowtie antenna array and a filled bowtie antenna array. (a) Spectrum of the 8 fs laser source. (b,c) Linear extinction (1 − *T*) spectra of the two arrays as shown in (d). (e,f) Second harmonic emission spectra of the two arrays. The increased signal strength observed for the filled antenna array is most likely caused by an increased spectral overlap between the antennas and the laser, rather than being due to the lithium niobate nanocrystals. Figure adapted from [2].

Figure 4 shows the results of linear and nonlinear measurements with both a filled nanoantenna array and a nanoantenna array without filling. Nanostructures were excited by 8 fs laser pulses centered at approximately 817 nm with a spectral bandwidth of 690 to 930 nm (VENTEON, pumped by a Coherent Verdi V10). The incoming radiation is focused onto the sample and recollected using spherical mirrors (*f* = 100 mm). The fundamental light is filtered out with a quartz glass prism sequence for measurement. The spectrally resolved detection is performed by a grating monochromater and an attached LN_2_-cooled UV-enhanced CCD camera sensitive to the fundamental as well as second harmonic light.

The laser spectrum is centered at a wavelength of 817 nm, as shown in Figure 4a. Figure 4b,c depicts the linear optical response of the antenna arrays, blue for the unfilled and in red for the filled antennas (SEM micrographs shown in Figure 4d). Figure 4e,f shows the second harmonic emission spectrum of the two arrays. On first sight one suspects that the increased second harmonic signal for the LiNbO_3_ filled antennas stems from the nanocrystals. However, several points have to be considered: First of all, the bowtie antenna array produces a second harmonic (SH) signal, which is symmetry forbidden in the lowest order, that is, in the electric dipole approximation. Second, we have not taken the modified linear optical properties into account. When examining Figure 4b,c we can clearly see that the linear extinction spectrum shifts so that the overlap between the driving laser source and the extinction increases. Therefore, it seems very likely that the increased SH response is caused by this shift rather than by an additional SH signal originating from the nanocrystals. The different peaks in the SH spectrum are caused by frequency mixing between the different spectral contributions of the fundamental laser spectrum.

**Figure 5:**
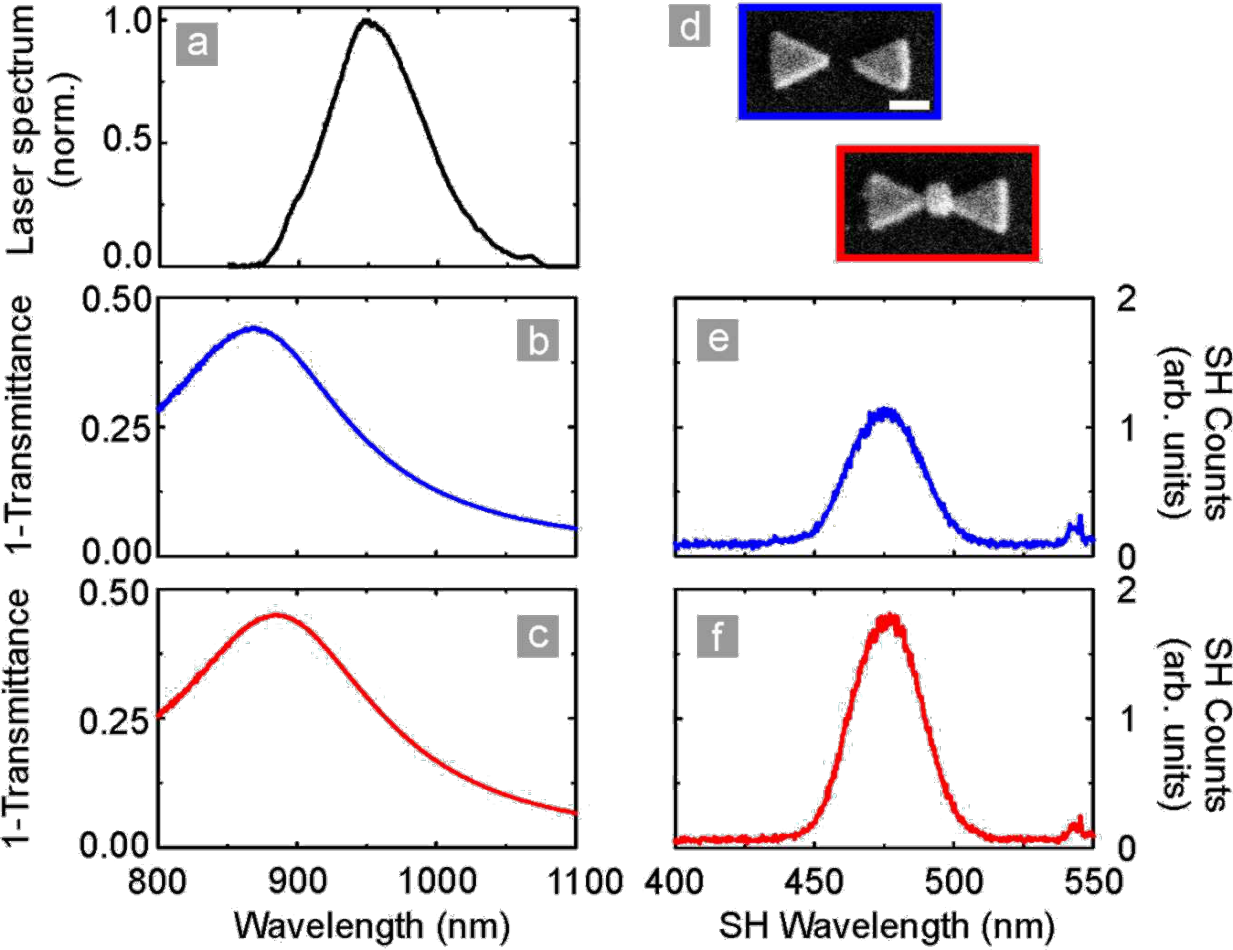
Linear and nonlinear properties of a bowtie antenna array and a filled bowtie antenna array. (a) Spectrum of the laser source. (b,c) Linear extinction (1 − *T*) spectra of the two arrays as shown in (d). (e,f) Second harmonic emission spectra of the two arrays. As in the previous case, one observes an increase in second harmonic signal; however, it is most likely caused by the increased spectral overlap, rather than by the nonlinear material deposited into the gap region. Figure adapted from [2].

The results shown in Figure 5 confirm this interpretation even further where the results have been obtained using a different laser source. We utilized a custom built Yb:KGW solitary mode-locked oscillator combined with a nonlinear photonic crystal fiber for spectral broadening. The pulses were subsequently sent into a 4*f* pulse shaper for amplitude and phase modulation, emitting 30 fs laser pulses tunable from 900 to 1180 nm. The radiated SH intensity is measured with a Peltier-cooled CCD camera [38,73]. The fundamental spectrum is shown in Figure 5a. As in the Figure 4, Figure 5b,c depicts the linear optical response of the antenna arrays, blue for the unfilled and in red for the filled antennas (SEM micrographs shown in Figure 5d). Figure 5e,f depicts the second harmonic emission spectrum of the two arrays. Yet again, the bare antenna array radiates second harmonic light, and the filled nanoantenna array produces a stronger signal. However, very similar to the previous case, the linear spectrum undergoes a spectral red-shift. This red-shift increases the overlap between the spectrum of the laser source and the linear extinction spectrum even stronger than in the case shown in Figure 4. Again, the increased signal is thus rather caused by a change in the linear response than by the insertion of the nanocrystals.

These findings indicate that we do not observe second harmonic generation from the nanocrystals. First of all, it is puzzling why the structures without the crystals already show a significant SH emission. In the electric dipole approximation we do not expect second harmonic emission from an inversion symmetric structure illuminated under normal incidence. However, strong SH emission from such structures has been reported [41]. Second, in both cases, the increase in signal strength can be solely explained by the increased spectral overlap of the laser source and the plasmon spectrum.

**Figure 6:**
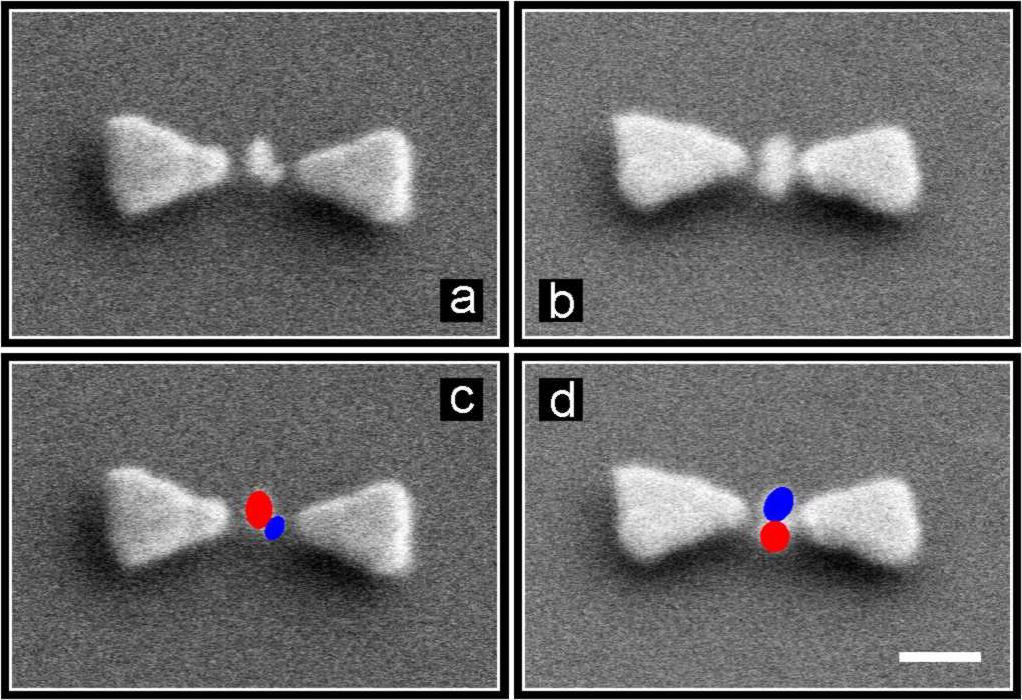
(a,b) Tilted view, close-up SEM images of two, exemplary, selectively filled, lithium niobate bowtie antennas. The antenna gaps contain several nanoparticles each, at least two, as indicated by the colored dots in the lower panels (c) and (d). The white scale bar is 100 nm. Figure adapted from [2].

There are actually several possible explanations for the observed behavior: Firstly, lithium niobate (under these circumstances) is a poor frequency converter. Lithium niobate exhibits an off-resonant nonlinearity which becomes efficient only for phase-matched geometries in bulk crystals of several mm in length. The absolute values of the χ^(2)^ tensor components are actually small when compared to those of gold. As the particles have a size of about 50 nm, they are extremely weak frequency converters. This conclusion is supported by the recent reports by Knabe et al. on the second harmonic emission from single lithium niobate nanocrystals, showing small conversion efficiencies under excitation with tightly focused, nanosecond laser pulses [71]. The nanocrystals have the same nonlinear optical coefficients as their bulk counterparts but evidently the effective nonlinear optical material volume is much smaller. Secondly, for the lithium niobate crystals, several χ^(2)^ tensor components are symmetry allowed. Therefore, it makes a huge difference how the particles are deposited inside the gap and in which direction they are aligned with respect to the antenna axis and thus relative to the polarization direction of the enhanced near-field. On the one hand, the absolute values of the components are very different, rendering the conversion efficiency strongly dependent on the orientation of the particles. On the other hand, the SH contributions from different nanocrystals are emitted with different phases, probably causing destructive interference of the SH emission from an ensemble of particles. Figure 6 depicts a tilted, close-up view of SEM images of filled bowtie antennas, clearly showing that more than one particle exists inside the gap (in the lower row the particles have been marked for clarity). Thirdly, recent reports suggest that the nonlinearity of gold is very strong. The emission of thin layers, boosted by the presence of a plasmon resonance, yields strong signals [40,44].

We wanted to suppress the strong nonlinearity of gold itself by rendering the antenna array centrosymmetric. No second harmonic signal should then be observable. Our findings seem to indicate a fundamental problem, as they suggest that symmetry considerations do not completely hold for our structures. This is possibly because the entire concept of tensorial nonlinear optics, based on effective media, does not hold, as our systems in fact consist of a 2D array of optical resonators of wavelength dimensions. Also, the structures exhibit significant surface roughness, which might be responsible for the observed signals. Such a signal would originate from a random effect (roughness) and is therefore only weak, if at all, linked to the geometry of the underlying array. A signal from the antennas themselves might therefore not be suppressible. One possible solution is the use of single crystalline, atomically flat, gold flakes fabricated via wet chemical synthesis and subsequent nanostructuring [74]. In order to increase a possible signal from the LiNbO_3_ particles, which intrinsically break inversion symmetry, we have to align them with respect to the antenna axis. As the particles are ferroelectric and exhibit a spontaneous macroscopic electric polarization, such an alignment can be accomplished by so-called corona poling [72]. When applying a strong external electric field to the particles they can be aligned with respect to the field and thus with respect to the antenna axis. However, as the particles already stick to the sample surface, it might not be possible to align them in a post-processing step. It is not yet clear if it is possible to accomplish the alignment while depositing the particles.

Nevertheless, our results suggest that the χ^(2)^ nonlinearity of lithium niobate might be too small in order to observe a strong SH emission from the hybrid system. The particles are only about 50 nm in diameter; hence the small conversion volume needs to be compensated by a significantly enhanced, fundamental field strength. However, the absolute value of the enhanced near-field is still highly debated and it is not yet fully understood what fundamentally limits the field strength and what values can actually be achieved. Ultimately, the electric field strength might be limited by either electron tunneling processes between the extremely close spaced metallic nanoparticles [75–76] or by so-called nonlocal effects where the dielectric function of the materials becomes wavevector and space dependent [77]. Yet, it seems to be commonly accepted that the initially proposed enhancements from theoretical and simulation studies of several orders of magnitude are not achievable in experiment. Moreover, the extremely high field strength is obviously only observed for extremely small gaps, that is, for gaps on the order of a few nanometers. Fabrication of plasmonic structures with such small gap sizes in a reliable and reproducible way is very challenging, and only a very limited number of publications so far have demonstrated the ability to achieve this goal. In all these cases, these techniques such as high-resolution electron beam lithography [78–79], self-assembled molecular monolayers [77], spacer layer engineering via atomic layer deposition [45], self-assembly of metallic nanoparticles with DNA and other molecular binding units [46,80–83] or by self-alignment of chemically synthesized metal particles [84] are very demanding. In any case, even if field enhancements of ≈80 might be achievable in Angstrom-scale gaps [76], the volume in which this field strength is present is extremely small, thus the volume of the active material will be small, too. Whether the extremely small amount of active material can be compensated by the high field strength is somewhat doubtful.

## Conclusion

The field of nonlinear plasmon optics is still in the very beginning stages. Even though the first experiments were already conducted in the early 1980s, several questions and problems remain unresolved. Surprisingly, this is often caused by the high complexity of the studied systems. This complexity is motivated by several reasons: Firstly, linear optical properties of these structures can be manipulated almost arbitrarily. Secondly, the structural geometry of the systems can be changed. This is supposed to be especially important because nonlinear optical processes are strongly dependent on symmetry. Thirdly, the dream to disentangle nonlinear and linear optical properties is a strong incentive for researchers, i.e., the hope to obtain strongly different nonlinear optical responses of two systems despite identical linear optical ones [43].

However, most of these properties are intimately connected and it is extremely difficult to disentangle the respective contributions. It is in particular important to realize the huge importance of the properties of the plasmonic resonances on the radiated nonlinear signals. The linewidth, representing the dephasing time and thus the time the energy is stored in the plasmonic cavity, is of particular importance. The nonlinear signal is thus extremely sensitive to seemingly minute changes in the linear optical properties of the plasmonic resonances. In fact, a number of experiments might actually be dominated entirely by the change in the linear optical properties rather than by the intended manipulation of, e.g., the symmetry of the system.

The dream, however, would be to go beyond this restriction and gain additional tunability, that is to have similar or even identical linear optical responses, yet the nonlinear optical properties would be strongly different. Only a limited number of experiments have demonstrated that the linear and nonlinear optical properties of a plasmonic or plasmon-hybrid system can indeed be decoupled. In all these cases, the cause for this deviation is actually the contribution of a dielectric medium, e.g., a waveguide. Utikal and co-workers demonstrated that the nonlinear response of different plasmon waveguide hybrid systems can be different in spite of nearly identical linear optical properties [61]. In this case, energy is transferred from the “bright” plasmonic resonance to the “dark” waveguide mode. The exact fraction of energy stored in the plasmon and waveguide modes is not encoded in the far-field spectra. Additionally, both systems have entirely different nonlinearities. Thus, the overall nonlinear response is determined by the relative near-field intensities and the relative strength of the nonlinearity in the two systems. Therefore, the nonlinear response cannot be predicted from the knowledge of the linear spectrum alone. Such behavior has probably not been demonstrated in a purely plasmonic system up to now, despite claims made.

From the conclusions above, it thus appears that the most promising route is the combination of plasmonically active components and dielectrics into hybrid structures, in particular for dielectric subsystems exhibiting strong nonlinear optical properties. As in the case of the nonlinear waveguide, the dielectric system does not manifest itself optically and thus does not allow for an accurate prediction of the nonlinear properties solely from the linear ones. The same holds for the nonlinear self-assembled monolayers on top of silver triangles [58]. Here, the monolayers are the source of the signal, rather than the silver triangles themselves. The main hurdle, however, is the weak nonlinearity of dielectrics when compared with noble metals, such as gold or silver. Nonlinear waveguides are thus a doubly smart choice, as the waveguide mode is an extended one and thus the interaction volume is large. The coupling of extended photonic modes to localized plasmonic modes hence seems appealing.

The field of nonlinear plasmon optics remains partially uncharted. With advances in sophisticated fabrication techniques for composite and hybrid structures, as well as advances in the rapidly growing field of theoretical and simulation-based descriptions of nonlinear plasmon optics, one can expect quite a number of fascinating discoveries in the next few years.
